# Reticulon 3 regulates sphingosine‐1‐phosphate synthesis in endothelial cells to control blood pressure

**DOI:** 10.1002/mco2.480

**Published:** 2024-02-13

**Authors:** Jie‐Yuan Jin, Si‐Hua Chang, Ya‐Qin Chen, Meng‐Wei Liu, Yi Dong, Ji‐Shi Liu, Qin Wang, Hao Huang, Liang‐Liang Fan, Rong Xiang

**Affiliations:** ^1^ Department of Orthopaedics Microsurgery & Reconstruction Research Center Xiangya Hospital Central South University Changsha China; ^2^ Department of Cell Biology, School of Life Sciences Central South University Changsha China; ^3^ National Clinical Research Center for Geriatric Disorders Xiangya Hospital, Central South University Changsha China; ^4^ Department of Cardiology The Second Xiangya Hospital of Central South University Changsha China; ^5^ College of Basic Medical Xinjiang Medical University Urumqi China; ^6^ Department of Nephrology The Third Xiangya Hospital of Central South University Changsha China

**Keywords:** ceramide synthase 2, ceramides, hypertension, hypotension, reticulon 3, sphingosine‐l‐phosphate

## Abstract

The discovery of the endothelium as a major regulator of vascular tone triggered intense research among basic and clinical investigators to unravel the physiologic and pathophysiologic significance of this phenomenon. Sphingosine‐l‐phosphate (S1P), derived from the vascular endothelium, is a significant regulator of blood pressure. However, the mechanisms underlying the regulation of S1P biosynthetic pathways in arteries remain to be further clarified. Here, we reported that Reticulon 3 (RTN3) regulated endothelial sphingolipid biosynthesis and blood pressure. We employed public datasets, patients, and mouse models to explore the pathophysiological roles of RTN3 in blood pressure control. The underlying mechanisms were studied in human umbilical vein endothelial cells (HUVECs). We reported that increased RTN3 was found in patients and that RTN3‐null mice presented hypotension. In HUVECs, RTN3 can regulate migration and tube formation via the S1P signaling pathway. Mechanistically, RTN3 can interact with CERS2 to promote the selective autophagy of CERS2 and further influence S1P signals to control blood pressure. We also identified an *RTN3* variant (c.116C>T, p.T39M) in a family with hypertension. Our data provided the first evidence of the association between RTN3 level changes and blood pressure anomalies and preliminarily elucidated the importance of RTN3 in S1P metabolism and blood pressure regulation.

## Graphical abstract

We first linked RTN3 with blood pressure regulation in humans and mice and reported the first *RTN3* variant in a hypertension patient. RTN3 controlled blood pressure partly through binding to CERS2 to induce its selective autophagy and negatively regulate S1P metabolism in endothelial cells. Our data preliminarily elucidated the importance of RTN3 in S1P synthesis and blood pressure regulation.

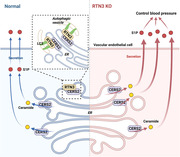

## 1 INTRODUCTION

Over recent years, significant evidence has accumulated indicating that patients with essential hypertension have an abnormality in endothelium function.^1^ Sphingosine‐l‐phosphate (S1P), a well‐known signaling molecule, regulates cell proliferation, motility, and survival.^2^ In blood vessels, S1P commands angiogenesis, vascular tension, and endothelial barrier stability.^3^ While major S1P is derived from erythrocytes, endothelial cells are also a major source.^4^ Endothelial cells secrete S1P, which applies to autologous S1P receptor 1/3 (S1PR1/3) to induce nitric oxide (NO) synthesis and secretion, increase cyclic guanosine monophosphate (cGMP) signaling, and then promote vasodilation.^5^ Conversely, higher concentrations of S1P result in vasoconstriction through S1PR2/3 on vascular smooth muscle cells (VSMCs).^6^

S1P can be produced by the S1P de novo biosynthetic pathway in the endoplasmic reticulum (ER), the salvage pathway in lysosomes, and sphingomyelin catabolism in the plasma membrane.^1^, ^7^ In these three synthetic pathways, ceramide is a crucial lipid molecule, which is catalyzed by ceramide synthases (CERSs).^7^, ^8^ In mammals, CERSs include six subtype proteins, CERS1‐6.^9^ Ceramides can be interconverted with sphingosine (Sph), which is further phosphorylated to S1P by sphingosine kinase 1 (SPHK1) or sphingosine kinase 2 (SPHK2).^8^ However, how S1P metabolism regulates the arterial system requires further clarification.

All S1P biosynthetic pathways originate from the membrane structure, and reticulon 3 (RTN3) is a classical membrane protein that is primarily distributed in the ER, Golgi apparatus, plasma membrane, and extracellular space.^8^, ^10^ Functionally, RTN3 may mediate vesicle transport, cellular communications, organelle contacts, autophagy, and lipid metabolism. For example, RTN3 is an ER–plasma membrane contact site that controls epidermal growth factor receptor nonclathrin endocytosis and regulates triglyceride metabolism by interacting with heat shock protein family A member 5 (HSPA5).^11^, ^12^, ^13^, ^14^ Whether RTN3 is involved in the metabolism of other lipids, such as ceramides and S1P, has not yet been investigated.

RTN3 has been identified in the mouse brain, kidney, liver, lung, testis, and ovary, especially the brain, and its aberrant expression is associated with obesity, hypertriglyceridemia (HTG), chronic kidney disease, and hepatocellular carcinogenesis.^10^, ^12^, ^15^, ^16^ Here, we identified the expression of RTN3 in thoracic aorta and vascular endothelium, provided the first evidence of the association between RTN3 level changes and blood pressure anomalies, and revealed that RTN3 maintains vascular homeostasis by negatively regulating S1P synthesis.

## 2 RESULTS

### 2.1 High expression of RTN3 is associated with arterial hypertension

**FIGURE 1:**
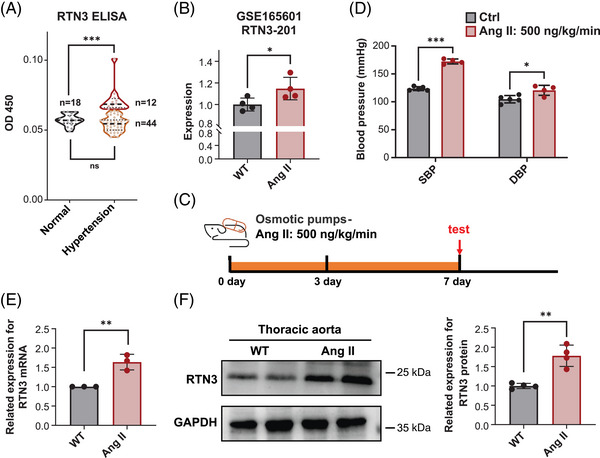
High expression of RTN3 is associated with high blood pressure. (A) Serum RTN3 levels were detected in healthy controls and hypertension patients. (B) The mRNA levels of RTN3 in wild‐type (WT) mice (*n* = 4) and angiotensin II (Ang II)‐treated mice (*n* = 4), based on GSE165601 data. (C) Schematic diagram of the mouse model. (D) Systolic blood pressure (SBP) and diastolic blood pressure (DBP) were detected in WT mice (*n* = 5) and Ang II‐treated mice (*n* = 4). (E) RTN3 mRNA levels in the thoracic aortas of WT mice (*n* = 3) and Ang II‐treated mice (*n* = 3). (F) RTN3 protein levels in the thoracic aortas of WT mice and Ang II‐treated mice were detected by western blotting (WB). “*” represents *p* ≤ 0.05; “**” represents *p* ≤ 0.01; “***” represents *p* ≤ 0.001.

Our previous study reported that overexpression of RTN3 may partly lead to HTG and obesity, which are important factors in the development of secondary hypertension.^12^, ^17^ To further investigate the association between RTN3 and hypertension, we enrolled 18 healthy controls and 56 primary hypertension patients with normal triglyceride levels (<1.7 mmol/L) and body mass index (18.5–24 kg/m^2^) to exclude the effects of HTG and obesity. Enzyme‐linked immunosorbent assay (ELISA) detection exhibited higher RTN3 levels in part patients (Figure 1A and Table 1).

**TABLE 1:** Clinical statistical data of patients.

Group	Control (*n* = 18)	Total hypertension (*n* = 56)	Hypertension with high RTN3 (*n* = 12)	Hypertension with normal RTN3 (*n* = 44)
Gender	18 M, 0 F	56 M, 0 F	12 M, 0 F	44 M, 0 F
Age (year)	34.444 ± 2.201	36.339 ± 1.165 (ns)	33.500 ± 2.201 (ns)	37.114 ± 1.330 (ns)
BMI (kg/m^2^)	22.350 ± 0.367	22.584 ± 0.181 (ns)	22.783 ± 0.361 (ns)	22.530 ± 0.209 (ns)
Triglyceride (mmol/L)	1.098 ± 0.088	1.080 ± 0.054 (ns)	1.099 ± 0.109 (ns)	1.074 ± 0.062 (ns)
RTN3 (OD 450)	0.057 ± 0.001	0.058 ± 0.001 (ns)	0.072 ± 0.003 (***)	0.054 ± 0.001 (ns)

BMI, body mass index; F, female; M, male; “ns,” no statistically significance, comparison with “Control”; “***,” *p* < 0.001, statistically significance, comparison with “Control.”

The GSE165601 database including the RNA‐seq data of ventral aortas from wild‐type (WT) mice (*n* = 4) and angiotensin II (Ang II)‐treated mice (*n* = 4) showed that the mRNA levels of RTN3 in Ang II‐treated mice were higher than those in WT mice (Figure 1B). To confirm this discovery, hypertension mouse models were constructed by Ang II osmosis pumps with a permeability of 500 ng/kg/min for 7 days, which is a classic hypertension modeling (Figure 1C). Both systolic blood pressure (SBP) and diastolic blood pressure (DBP) were obviously increased in Ang II‐treated mice compared with saline‐treated mice (Figure 1D). Real‐time PCR and western blot (WB) authenticated the increased RTN3 in thoracic aortas of Ang II‐treated mice (Figures 1E and F). These discoveries suggested the association between high expression of RTN3 and hypertension and that this linkage may be independent of obesity and HTG.

### 2.2 RTN3‐null mice present hypotensive conditions

**FIGURE 2:**
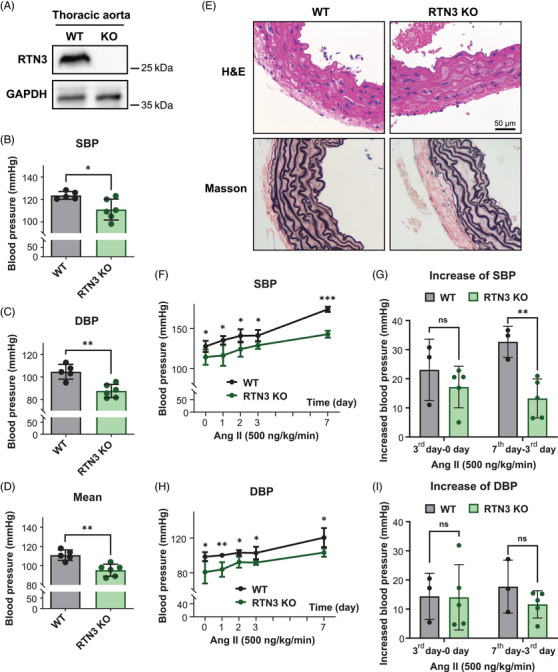
RTN3‐null mice present lower blood pressure. (A) RTN3 protein levels in the thoracic aortas of WT mice and RTN3‐null mice were detected by WB. (B–D) SBP (B), DBP (C), and mean arterial pressure (D) were detected in WT mice (*n* = 5) and RTN3‐null mice (*n* = 6). (E) H&E and Masson staining showed the incompact elastin fibers of aortas in the RTN3‐null mice. (F) Time course of SBP in WT mice (*n* = 3) and RTN3‐null mice (*n* = 5) treated with Ang II. (G) The incremental SBP after implantation of an osmotic pump delivering Ang II in WT mice (*n* = 3) and RTN3‐null mice (*n* = 5). (H) Time course of DBP in WT mice (*n* = 3) and RTN3‐null mice (*n* = 5) treated with Ang II. (I) The incremental DBP after implantation of an osmotic pump delivering Ang II in WT mice (*n* = 3) and RTN3‐null mice (*n* = 5). “ns” represents *p* > 0.05; “*” represents *p* ≤ 0.05; “**” represents *p* ≤ 0.01; “***” represents *p* ≤ 0.001.

We have reported that RTN3 overexpression is associated with HTG and obesity, which are important factors for secondary hypertension. We then generated RTN3‐null mice as we previously described to continue the study.^12^ We identified that RTN3 was expressed in thoracic aortas but silenced in RTN3‐null mice (Figure 2A). As expected, compared with WT mice, RTN3‐null mice were obviously hypotensive, and their SBP, DBP, and mean arterial pressure were significantly lower (Figures 2B–D). Hematoxylin–eosin (H&E) and Masson staining showed the incompact elastin fibers of aortas in the RTN3‐null mice (Figure 2E).

Next, we applied Ang II (500 ng/kg/min) to WT and RTN3‐null mice for 7 days. The RTN3‐null mice presented lower SBP and DBP than WT mice after Ang II treatment at all observation times (Figures 2G and H). Simultaneously, the increase in SBP on the 7th day after Ang II treatment in RTN3‐null mice was also less than that in WT mice (Figures 2I and J). Our findings revealed that RTN3 triggered hypotension in mice and may attenuate Ang II‐induced elevated blood pressure.

### 2.3 RTN3 reduction promotes the migration and tube formation of HUVECs

VSMCs and endothelial cells are the two most dominant classes of cells in thoracic aortas.^18^ First, we analyzed the effects of RTN3 reduction on VSMCs. We stripped the mouse aortic media in RTN3‐null mice or transfected RTN3 siRNA into human aortic VSMCs (HA‐VSMCs) and then tested vinculin, actin alpha 2, smooth muscle (α‐SMA), calponin 1 (CNN1), and calponin 2 (CNN2), which are associated with blood pressure regulation or phenotypic switch of VSMCs.^19^, ^20^, ^21^ However, we did not observe prominent alterations in the expression of these proteins (Figures S1A and B). Transwell results indicated that reduced RTN3 did not disturb HA‐VSMC migration (Figure S1C). This result suggested that the lack of RTN3 had very little effect on VSMCs.

**FIGURE 3:**
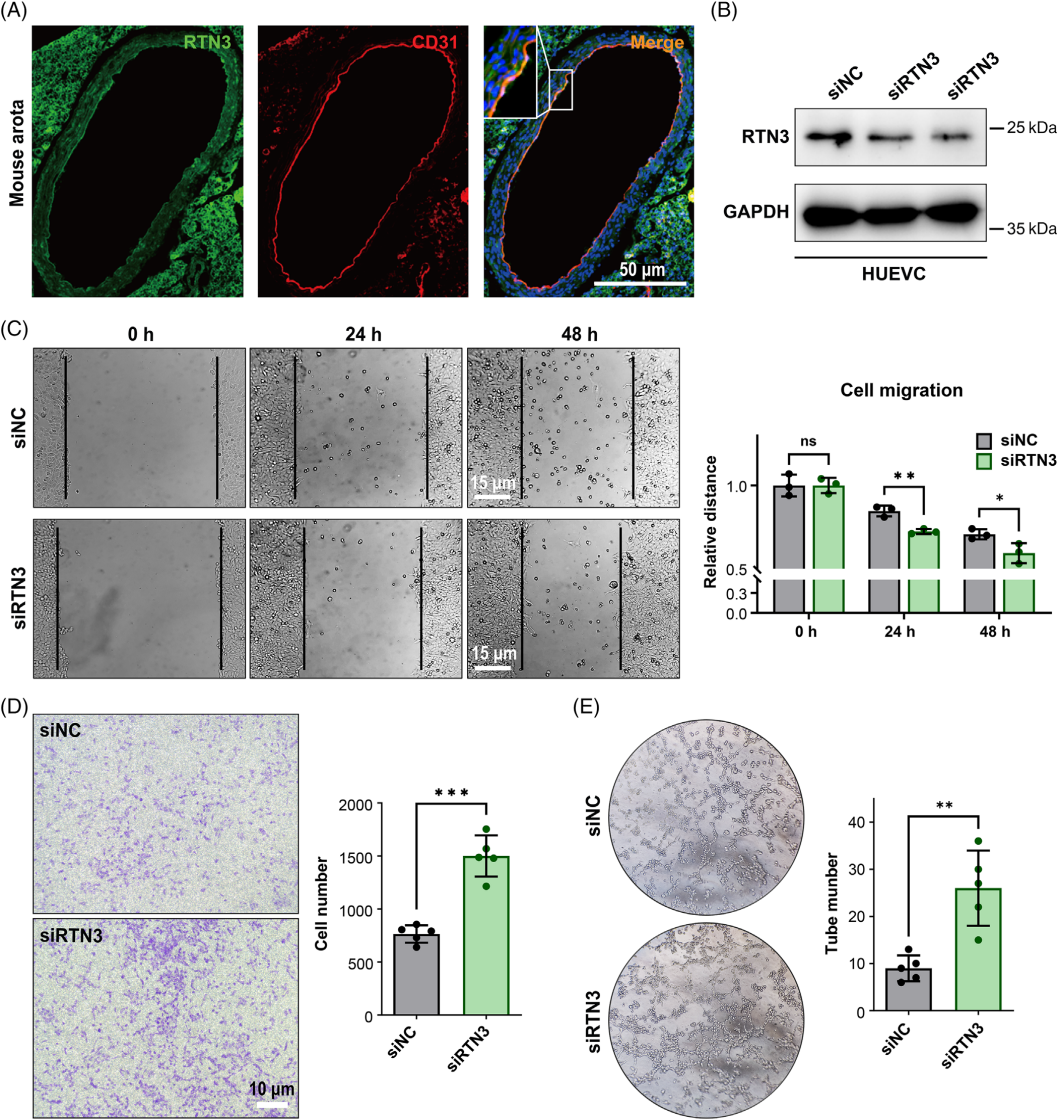
Reduced RTN3 facilitates cell migration and cell tube formation in HUVECs. (A) RTN3 was found to be expressed in vascular endothelial cells using confocal microscopy. (B–G) RTN3 protein levels (B), cell migration (C and D), and cell tube formation (E) in HUVECs transfected with RTN3 siRNA and controls, as detected by western blotting (WB), the scraping line method, transwell, and tube formation assays. “ns” represents *p* > 0.05; “*” represents *p* ≤ 0.05; “**” represents *p* ≤ 0.01; “***” represents *p* ≤ 0.001.

Hence, we then focused on the effect of RTN3 reduction on endothelial cells. Immunofluorescence staining found that RTN3 colocalized with the endothelial cell marker CD31 (platelet and endothelial cell adhesion molecule 1) in WT mouse thoracic aortas, which suggested that RTN3 was expressed in endothelial cells (Figure 3A). To assess the effect of RTN3 reduction in endothelial cells, we transfected RTN3 siRNA into human umbilical vein endothelial cells (HUVECs; Figure 3B). The scraping line method and transwell studies revealed that knocking down RTN3 expression in HUVECs may promote cell migration ability (Figures 3C and D). Cell tube formation manifested that silencing the expression in HUVECs may increase the angiogenesis of cells (Figure 3E). In addition, siRTN3 suppressed the transition from G1 to S phase but did not induce cell apoptosis to reduce cell viability in HUVECs (Figure S2). Together, these findings suggested that downregulated RTN3 can promote the migration and tube formation of HUVECs.

### 2.4 Lack of RTN3 activates S1P the signaling pathway

**FIGURE 4:**
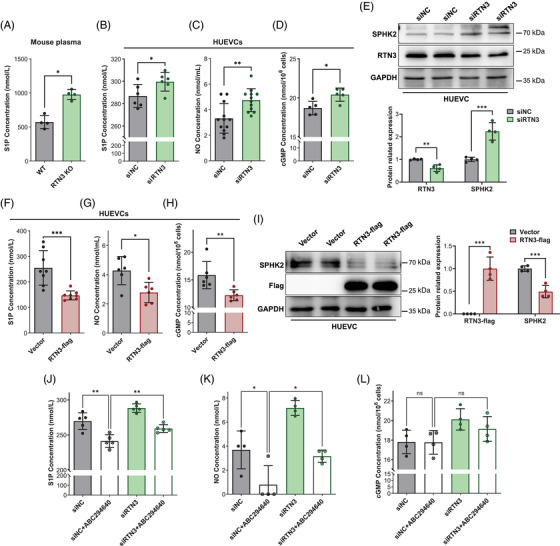
Lack of RTN3 activates the S1P signaling pathway. (A) plasma sphingosine‐1‐phosphate (S1P) levels in WT mice and RTN3‐null mice. (B–D) Extracellular S1P (B), extracellular NO (C), and intracellular cGMP (D) in HUVECs transfected with RTN3 siRNA and controls. (E) The expression of RTN3 and SPHK2 in HUVECs with/without RTN3 siRNA transfection. (F–H) The levels of extracellular S1P (F), extracellular NO (G), and intracellular cGMP (H) in HUVECs overexpressing RTN3 and controls. (I) The expression of RTN3 and SPHK2 in HUVECs with over‐expressed RTN3 and controls. (J–L) The levels of extracellular S1P (J), extracellular NO (K), and intracellular cGMP (L) in HUVECs transfected with siRTN3 and/or treated with ABC294640. “ns” represents *p* > 0.05; “*” represents *p* ≤ 0.05; “**” represents *p* ≤ 0.01; “***” represents *p* ≤ 0.001.

Previous studies have claimed that S1P plays a significant role in blood pressure regulation, cell migration, and tube formation of endothelial cells.^22^ We detected the S1P levels in the mouse models. S1P from the plasma of RTN3‐null mice was markedly increased (Figure 4A). However, the S1P levels in the aortas of RTN3‐null mice and WT mice were not significantly different (Figure S3A). We speculated that the majority in the arteries were tunica media and that S1P from tunica media/VSMCs covered the difference in S1P from the endothelium.

Then, we assessed S1P levels in HUVECs. The cell supernatant of HUVECs transfected with siRTN3 contained more S1P than that of the controls (Figure 4B). Simultaneously, HUVECs lacking RTN3 were accompanied by the increase in extracellular NO and intracellular cGMP, which may be induced by S1P signaling (Figures 4C and D). Moreover, WB analysis found that the sphingosine kinase isozyme SPHK2, but not SPHK1, which catalyzed the phosphorylation of sphingosine into S1P, was increased in HUVECs with RTN3 knockdown (Figures 4E and S3B).^8^ However, similar to S1P in aortas, both SPHK1 and SPHK2 were also not discriminative in RTN3‐null mouse aortas and WT mouse aortas (Figures S3C and D). Conversely, an overabundance of RTN3 in HUVECs caused diminished S1P, NO, cGMP, and SPHK2 levels (Figures 4F–I). Thus, we reasoned that lack of RTN3 can activate the S1P signaling pathway to lower blood pressure and stimulate the migration and tube formation of endothelial cells.

3‐(4‐Chlorophenyl)‐adamantane‐1‐carboxylic acid (pyridin‐4‐ylmethyl) amide, named ABC294640, is a selective inhibitor of SPHK2 that can decrease S1P synthesis and rescue Ang II‐induced hypertension in mice.^23^, ^24^ Here, ABC294640 and/or RTN3 siRNA were applied to treat HUVECs, and the results showed that the levels of S1P and NO after ABC294640 treatment were lower than those in the controls, while compared with the siN+ABC294640 group, they were obviously increased in the siRTN3+ABC294640 group (Figures 4J–L). These studies suggested that silencing RTN3 expression in HUVECs can attenuate the inhibitory effects of ABC294640 on S1P biosynthesis. All these findings suggested that reducing RTN3 activated the S1P signaling pathway in HUVECs.

### 2.5 RTN3 interacts with CERS2 to play a negative role in ceramide synthesis

**FIGURE 5:**
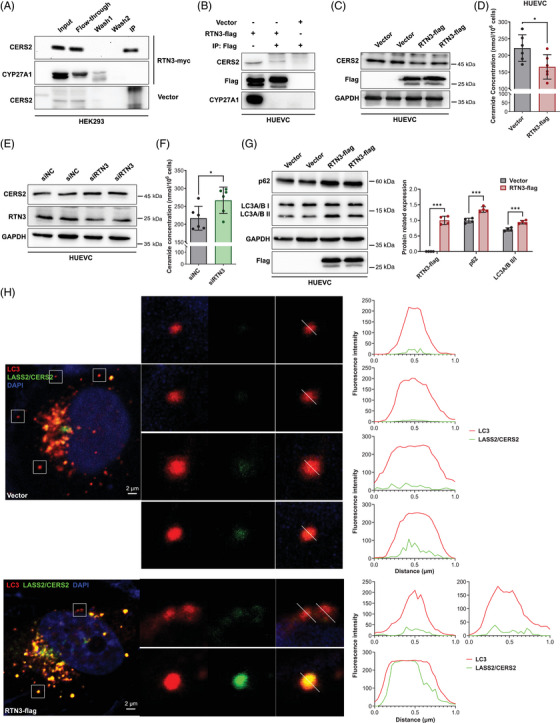
RTN3 interacts with CERS2 to promote CERS2 selective autophagy and plays a negative role in ceramide synthesis. (A and B) Coimmunoprecipitation (Co‐IP) confirmed that RTN3 can interact with CERS2 in HEK293 cells (A) and HUVECs (B). (C) The protein levels of RTN3‐myc and CERS2 in HUVECs transfected with RTN3 and controls. (D) The intracellular ceramide levels in HUVECs overexpressing RTN3 and the control group. (E) The protein levels of RTN3 and CERS2 in HUVECs transfected with isRTN3 and controls. (F) The intracellular ceramide levels in controls and HUVECs transfected with isRTN3. (H) The protein levels of RTN3‐myc, p62, and LC3A/B I/II in HUVECs transfected with RTN3 plasmids and controls after fasting for four hours. (I) Confocal microscopy found that CERS2 (green) showed more colocalization with LC3A/B (red) in HUVECs transfected with RTN3 overexpression plasmids than in cells transfected with blank vector. “*” represents *p* ≤ 0.05; “***” represents *p* ≤ 0.001.

We found that RTN3 can regulate the S1P pathway in HUVECs, which plays a crucial role in blood pressure regulation, but the underlying molecular mechanisms are unclear. Mass spectrometry was applied to detect the candidate RTN3‐interacting proteins in HUVECs. Interestingly, CERS2, a ceramide synthase and a core enzyme in S1P synthesis pathways, was found to be a possible RTN3‐interacting protein (data not shown).^7^ Coimmunoprecipitation (Co‐IP) analysis further validated that CERS2 can interact with RTN3 in human embryo kidney 293 (HEK293) cells and HUVECs (Figures 5A and B). CERS2 predominated in mouse aortas, as detected by unique identifier‐mRNA sequencing (Figure S4A). In addition, CERS1, which was slightly expressed in mouse aortas, also interacted with RTN3 in HEK293 cells and HUVECs (Figures 4B and C). WB analysis revealed that overexpressed RTN3 impeded CERS1 in HEK293 and CERS2 in HUVECs (Figures 5C and S4D). Correspondingly, the downstream total ceramide (intracellular) level was also reduced (Figures 5D and S4E). In contrary, the expression of CERS2 and the concentration of intracellular ceramides increased when HUVECs were transfected with siRTN3 (Figures 5E and F).

However, why can RTN3 regulate the expression of CERS2? By reviewing literatures, we found that RTN3 has been reported to be involved in the formation of autophagic vesicles.^13^ WB validated that autophagy markers, autophagy‐related protein LC3 A and B (LC3A/B II) and autophagy receptor P62 (p62), were more highly expressed in HUVECs with increased RTN3 after starvation for four hours (Figure 5G). Confocal microscopy found that only a little LC3A/B did not colocalized with CERS2 in HUVECs transfected with RTN3 overexpression plasmids, less than cells transfected with blank vector (Figure 5H), which indicated that autophagic vesicles were easier to degrade the CERS2 when RTN3 increased in HUVECs. In fact, RTN3 is one of binding sites of LC3 in ER, to induce the formation of autophagic vesicles.^25^ CERS2, as an interacting protein of RTN3, may be more liable to be parceled into autophagic vesicles.

**FIGURE 6:**
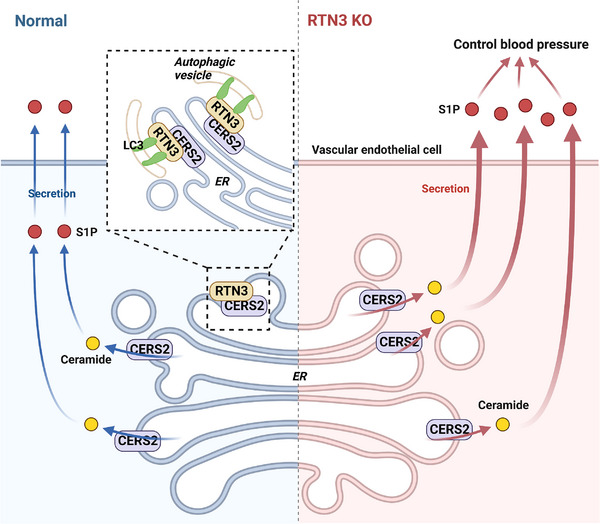
Potential mechanism by which RTN3 affects the biosynthesis of ceramide and S1P to control blood pressure. RTN3 is one of binding sites of LC3 in endoplasmic reticulum, to induce the formation of autophagic vesicles. RTN3 can interact with CERS2 in endothelial cells to make it more liable to be parceled into autophagic vesicles and degradation. However, RTN3 reduction can retain more CERS2 to stimulate the synthesis of ceramide and S1P, and the imbalance of S1P metabolism would lead to blood pressure variation. The figure was created with BioRender.com.

Collectively, our data suggested that RTN3 can interact with CERS2 in endothelial cells. As a marker of cell autophagy, RTN3 may promote the formation of autophagic vesicles, and more CERS2 was bound by RTN3 into autophagic vesicles to be degraded. As a ceramide synthase, reducing CERS2 may decrease ceramide to further inhibit S1P produce. However, reduced RTN3 correspondingly retained more CERS2 to stimulate the synthesis of ceramide and S1P. The imbalance of S1P metabolism would lead to blood pressure variation (Figure 6).

### 2.6 An *RTN3* variant (c.116C>T, p.T39M) is identified in a patient with hypertension

**FIGURE 7:**
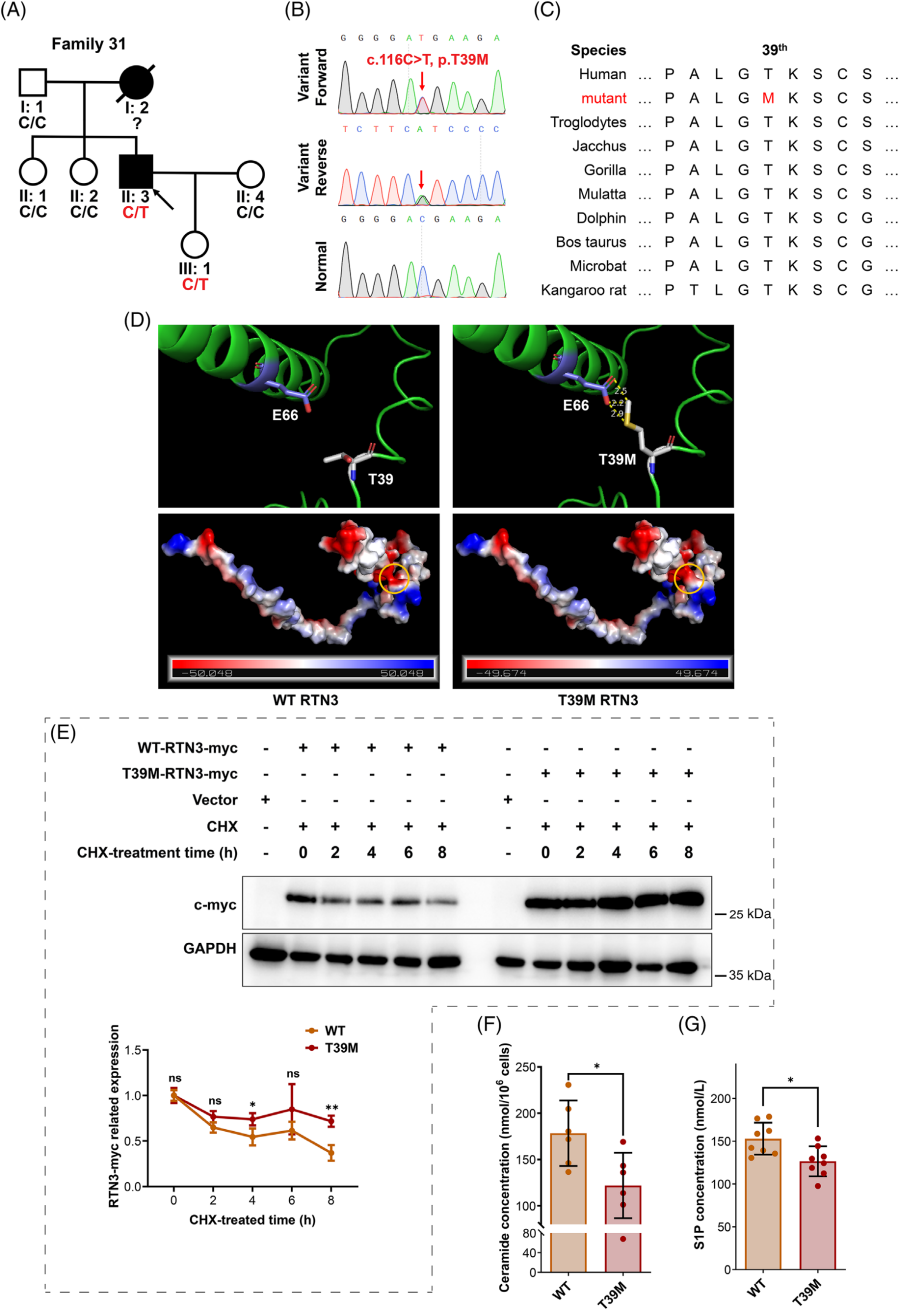
An *RTN3* variant (c.116C>T, p.T39M) is identified in a patient with hypertension. (A) The family pedigree of Family 31. (B) Sanger sequencing results of the *RTN3* variant (c.116C>T, p.T39M). (C) Species conservation analysis of the mutant amino acid sites of RTN3. (D) Three‐dimensional model of the human WT RTN3 protein fragment and p.T39M mutant protein. (E) The protein levels of RTN3 and mutant RTN3 T39M in HUVECs treated with cyclohexane (CHX). (F and G) The intracellular ceramide (F) and extracellular S1P levels (G) in HUVECs transfected with WT RTN3 plasmids or p.T39M mutant RTN3 plasmids. “ns” represents *p* > 0.05; “*” represents *p* ≤ 0.05; “**” represents *p* ≤ 0.01.

Additionally, 48 hypertensive families were enrolled to perform mutation detection by whole‐exome sequencing (WES). An *RTN3* variant (c.116C>T, p.T39M) was identified in Family 31 (Figures 7A and B). The proband (II:3) was a 42‐year‐old male with SBD of 150 mmHg and PBD of 110 mmHg. He was diagnosed with hypertension 6 years ago, and his mother (I:2) also had hypertension with the onset of her forties. He and his daughter (III:1) harbored the *RTN3* variant, while the girl was unaffected thus far (Table 2). Bioinformatics analysis manifested that p.T39 in RTN3 was highly conserved in mammals, especially primates (Figure 7C). Three‐dimensional protein modeling revealed that the variant p.T39M sharply reduced the distance between the 39th amino acid and the 66th (p.E66), possibly changing the spatial structure of RTN3, and that the substitution of positively charged threonine by noncharged methionine moderately altered the surface charge of RTN3 (Figure 7D).

**TABLE 2:** The phenotypes of the affected or *RTN3*‐variant carriers in family F31.

Patients	Gender	Age (year)	Onset age (year)	Phenotypes	Genotype
I:2 mother	F	62 (death)	>40	Hypertension	Unknown
II:3 proband	M	42	<36	SBP: 150 mmHg, DBP: 110 mmHg; BMI: 23.7 kg/m^2^; Tg: 1.64 mmol/L	*RTN3* variant: c.116C>T, p.T39M
III:1 daughter	F	16	–	SBP: 107 mmHg, DBP: 78 mmHg; BMI: 20.5 kg/m^2^; Tg: 1.52 mmol/L	*RTN3* variant: c.116C>T, p.T39M

BMI, body mass index; DBP, diastolic blood pressure; F, female; M, male; SBP, systolic blood pressure; Tg, triglycerides.

To verify the pathogenicity of the *RTN3* variant, we constructed WT and RTN3 mutated (p.T39M) plasmids. We transfected WT and mutant plasmids into HUVECs, and after treatment with cyclohexane (CHX), RTN3 mutant protein presented higher related expression than WT RTN3, suggesting that the variant p.T39M made RTN3 more stabilized and resistant to degradation (Figure 7E). Ceramide and S1P levels were also reduced in the mutated RTN3 group (Figures 7F and G). Thus, we reasoned that the *RTN3* variant (c.116C>T, p.T39M) is one of the etiological factors in the proband.

## 3 DISCUSSION

Hypertension is one of the most common diseases with one billion people affected, and its incidence is increasing year by year.^26^ Despite its prevalence, the cellular and molecular mechanisms of hypertension remain poorly understood, highlighting the need for further research on its pathogenesis. Over the past years, significant evidence has accumulated indicating that patients with essential hypertension have an abnormality in endothelium‐dependent vasodilator function.^1^, ^7^ In this study, we found that RTN3 can regulate S1P synthesis in endothelial cells to control blood pressure.

Our previous study revealed that overexpressed RTN3 is associated with obesity and HTG, which are connected with hypertension.^12^ Exceeding our estimation, the increased expression of RTN3 was also detected in subjects with primary hypertension but without obesity and HTG. For these high‐RTN3‐expression patients but without obesity and HTG, we thought that it may be caused by individual differences, environments, dietary habits, and/or life styles. In addition, Ang II‐induced hypertensive mice without obesity and HTG also appeared increased RTN3. RTN3‐null mice were hypotensive. These findings suggested that the connection between RTN3 and hypertension is not only dependent on obesity and HTG. Thus, we considered that in this study, mice lacking RTN3 are more appropriate to investigate the association with RTN3 and blood pressure regulation. However, we only recruited 56 hypertensive subjects, and given that sex chromosomes and sex hormones influence the differences in blood pressure regulation, cardiovascular‐disease risks, and comorbidities in females and males with essential arterial hypertension, we did not recruit female subjects.^27^ In further investigation, more participants, especially females, can contribute to comprehending the correlation between RTN3 and hypertension.

S1P activates NO to exert regulatory effects in the vasculature, including angiogenesis, vascular tone, and endothelial barrier integrity.^28^ Vessel endothelium‐originated S1P enables the S1P signaling pathway in endothelial cells through autocrine and paracrine mechanisms.^29^ In this study, facilitated S1P signaling could result as a consequence of silenced RTN3 within HUVECs and thereby accelerate cell migration and angiogenesis. Ceramide is a core lipid molecule in all S1P biosynthesis pathways.^8^ Different CERSs prefer different acyl chain coenzyme A, such as CERS1 catalyzing C18 (dihydro) ceramide, and CERS2 synthesizing ceramides containing C20:0, C22:0, C24:1, C24:0, C26:1, and C26:0 fatty acids.^30^, ^31^ In this study, RTN3 was observed to interact with CERS1 and CERS2, estimated to widely effect the synthesis of multiple ceramides. Considering the effects of RTN3 on autophagy, we further confirmed that increased RTN3 promoted CERS2 selective autophagy and then reduced the levels of ceramide and S1P in HUVECs, which may disrupt endothelial barrier function and ultimately induce hypertension. In contrast, RTN3 absence enhanced intracellular ceramide, and then SPHK2 was passively augmented to alleviate ceramide increase and induce S1P generation to trigger hypotension.^32^, ^33^ Thus, we reasoned that RTN3 regulates S1P synthesis through interaction with CERS1/2 to control blood pressure.

In addition, we identified an *RTN3* variant (c.116C>T, p.T39M) in a hypertensive family. This variant was detected to reduce ceramide and S1P in HUVECs and may be the first *RTN3* variant reported in hypertension. Given that hypertension is not a disease with onset at an early age, we considered it natural that the young *RTN3*‐variant carrier was unaffected. Her father, the proband, was also diagnosed with hypertension in his thirties. In fact, dysfunction of the endothelium is associated with hypertension and probably precedes its development.^34^ We speculated that the *RTN3* variant inhibited protein degradation and that RTN3 accumulation disrupted the balance of sphingolipid metabolism, triggering hypertension. The family members will continue to be followed up. A few *RTN3* variants were also identified in Alzheimer's disease; however, authors did not pay attention to patients’ blood pressure.^35^

In this study, we believed that RTN3 regulated vascular homeostasis mainly depending on its effects on the endothelium rather than vascular α‐SMA, which should still be rigorously confirmed in mice lacking RTN3, specifically in endothelial cells or VSMCs. Due to the lack of inhibitors and antagonists for RTN3, rescue experiments have not been conducted. The investigation into related small molecule drugs can improve the reliability of our findings and may be developed into an alternative treatment for hypertension in the future.

In addition to S1P, ceramides can also be used to fabricate cerebroside, sphingomyelin, glycosphingolipid, and 1‐phosphonate ceramide, and in theory, with the increase in ceramides triggered by RTN3 deficiency, other downstream substances would also be augmented.^7^ However, sphingomyelin and ceramides themselves were reported to be linked with hypertension.^36^, ^37^ Moreover, both RTN3 deficiency and ABC294640 can reduce blood pressure in Ang II‐treated mice, but ABC294640 restrained S1P, while RTN3 absence induced S1P.^24^ These findings suggested the complexity of the regulation of blood pressure accommodated by RTN3, which should be investigated in further studies.

## 4 CONCLUSIONS

In summary, we provided the first evidence on the association between RTN3 and blood pressure regulation in humans and mice. High RTN3 expression was accompanied by hypertension, and the absence of RTN3 resulted in hypotension in mice. We reported the first *RTN3* variant (c.116C>T, p.T39M) identified in a hypertension patient to further confirm this correlation. RTN3 controlled blood pressure partly through binding to CERS2 to induce its selective autophagy and negatively regulate S1P metabolism in endothelial cells. Overall, our data preliminarily elucidated the importance of RTN3 in blood pressure regulation and emphasized the causative role of S1P signal dysregulation in endothelium dysfunction and hypertension.

## 5 MATERIALS AND METHODS

### 5.1 Subject recruitment

A total of 18 controls and 56 hypertension patients were recruited. All subjects were male with serum triglycerides < 1.7 mmol/L, 18.5 kg/m^2^ < BMI < 24 kg/m^2^, and they ranged in ages from 20 to 50. All subjects or their guardians consented to publication of the clinical details.

### 5.2 Mouse strains and modeling

RTN3‐null mice were generated, and their genotypes were identified as described previously.^12^ C57BL/6J mice were purchased from the Chinese Academy of Sciences (Shanghai, China) and bred in the Department of Zoology, Central South University.

Ang II (Solarbio, Beijing, China) was induced in male mice at 10 weeks of age. Ang II powder was diluted to an appropriate concentration using sterile normal saline and injected into Alzet implantable osmotic pressure capsules (Durect, Cupertino, USA) with a special needle, and the penetration rate was 500 ng/kg/min^6^. Alzet capsules were inserted into the mouse dermis, and the wound was sutured. A Coda Non‐Invasive Blood Pressure System (Kent Scientific, Torrington, USA) was used to detect the noninvasive blood pressure in the tail vein of mice.

### 5.3 GSE165601 database analysis

The RNA‐seq datasets of GSE165601 were obtained from the GEO database (https://www.ncbi.nlm.nih.gov/geo/query/acc.cgi?acc=GSE165601). R (version 4.0.4) and RStudio (version 1.2.5033) were used to address all the data in this study. GSE185051 contained the expressed genes in the abdominal aorta of five C57BL/6 WT mice and five Ang II‐treated mice.

### 5.4 H&E staining and Masson staining

Paraformaldehyde‐fixed aortas were embedded in paraffin and sliced into 5 μm sections. The sections were dried, dewaxed with xylene and rehydrated with decreasing concentrations of alcohol. For H&E staining, slides were stained with hematoxylin (Beyotime, Shanghai, China) for 15 min, and stained with 1% eosin (Beyotime) for 2 min. Masson's trichrome reagent (Solarbio) was used for Masson staining.^38^

### 5.5 Cell culture and transfection

The pcDNA3.1‐flag‐RTN3 plasmid and pcDNA3.1‐myc‐RTN3 plasmid were purchased from Sangon Biotech Company (Shanghai, China). Mutagenesis of RTN3 c.116C>T (pcDNA3.1‐flag‐mutRTN3‐T39M) was performed using Fast Mutagenesis Kit V2 (Vazyme, Nanjing, China).

HUVECs and HEK293 cell lines were obtained from the Cell Bank of Shanghai Institutes for Biological Sciences (Shanghai, China). HUVECs were cultured in Roswell Park Memorial Institute (RPMI‐1640) medium, and HEK293 cells were cultured in Dulbecco's modified Eagle's medium. HUVECs were transfected with the pcDNA3.1‐flag‐RTN3 plasmid. Cells were seeded in 6‐well plates and transfected with RTN3 siRNA (RiboBio, Guangzhou, China) or plasmids for 24 h to operate the scraping line method, transwell, cell tube formation, and 60 μM ABC294640 (APExBIO, Houston, USA) treatment with test after 24 h, or for 48 h to operate WB, ELISA, Co‐IP, 100 μM CHX (Solarbio) treatment, and flow cytometry.

### 5.6 WB, Co‐IP, and confocal

Proteins were extracted by ristocetin‐induced platelet agglutination buffer (Solarbio), and WB, Co‐IP, and confocal microscopy were performed as previously described.^12^ Anti‐RTN3 primary antibody (1:2000) was self‐produced. Anti‐GAPDH primary antibody (10494‐1‐AP, 1:5000), anti‐flag primary antibody (20543‐1‐AP, 1:5000), and anti‐p62 primary antibody (18420‐1‐AP, 1:1000) were purchased from Proteintech Company (Wuhan, China). Anti‐SPHK2 primary antibody (YT4383, 1:1000) and anti‐CYP27A1 primary antibody (YT1202, 1:1000) were purchased from ImmunoWay Biotechnology Company (Suzhou, China). Anti‐myc primary antibody (sc‐40, 1:100), anti‐CERS2 primary antibody (sc‐390745, 1:100), and anti‐LC3A/B primary antibody (sc‐398822, 1:100) were purchased from Santa Cruz Biotechnology Incorporated (Santa Cruz, USA). Anti‐myc magnetic beads (Beyotime) and anti‐flag magnetic beads (Beyotime) were used for Co‐IP, and self‐produced anti‐RTN3 primary antibody (1:400) and anti‐CD31 primary antibody (66065‐2‐Ig, 1:200) were purchased from Proteintech Company; anti‐LC3 primary antibody (PM036, 1:200) were purchased from MBL International Corporation (Kyushu, Japan); anti‐CERS2 primary antibody (sc‐390745, 1:100) and DAPI (Solarbio) were used for confocal microscopy.

### 5.7 ELISA and NO assay

Human plasma was isolated from blood samples collected by coagulation promoting. Plasma RTN3 levels were tested by self‐produced RTN3 ELISA embedding assay. Eight microliters of plasma and 92 μL embedding buffer (2.93 g NaHCO_2_ and 1.59 g Na_2_CO_2_ dissolved in ultrapure water) were embedded in 96‐well plates, washed, blocked, and incubated with primary antibody and secondary antibody, and ultimately, 3,3′,5,5′‐tetramethylbenzidine substrate (Solarbio) and stop buffer were added to measure in 450 nm absorbance.

S1P was tested in mouse plasma isolated from blood sampled by eyeball extirpating, in the aortic tissue homogenate of mice dissolved in PBS, and in the supernatant from cells cultured in complete medium for 48 h using S1P ELISA assay kits (Mlbio, Shanghai, China). A NO assay kit (Beyotime) was used to detect the NO content in the culture supernatant. The cell number was counted by a cell counter. Cells were collected, dissolved in PBS, and lysed by freeze–thaw cycles. The lysates were used to detect ceramide and cGMP using ceramide and cGMP ELISA assay kits (Mlbio). Details are provided in the Supplementary Materials and Methods.

### 5.8 Scraping line method, transwell, and cell tube formation assay

After transfection of siRTN3 into HUVECs for 24 h, a straight line was drawn in each well of 12‐well plates, where the change in width was recorded every 24 h. Five thousand to ten thousand cells were seeded in transwell (Costar, Suzhou, China) and cultured for 24 h to check cell migration. Fifteen thousand cells were seeded in Matrigel (Corning, Corning, USA) for 3 h to check cell tube formation.

### 5.9 Real‐time PCR

Total RNA was extracted using an RNA Extraction Kit (Qiagen, Dusseldorf, Germany) and stored at −80°C. Mouse RTN3 and GAPDH primer pairs (mouse RTN3 real‐time PCR f: 5′‐GGTAGAAGACTTGGTTGACTCC‐3′; mouse RTN3 real‐time PCR r: 5′‐GGCGAGAATCAGAAGGGTAAT‐3′; mouse GAPDH real‐time PCR f: 5′‐CCCTTCATTGACCTCAACTACA‐3′; mouse GAPDH real‐time PCR r: 5′‐CCCTTCATTGACCTCAACTACA‐3′) were designed. Total RNA was reverse transcribed using a RevertAid First Strand cDNA Synthesis Kit (Thermo, Waltham, USA) and then subjected to real‐time PCR with 2xSYBR Green qPCR Mix (Thermo).

### 5.10 Genetic screening

Genomic DNA was extracted using the DNeasy Blood & Tissue Kit (Qiagen, Valencia, USA). WES was performed by Berry Genomics Company (Chengdu, China) using the Illumina HiSeq4000 platform (Illumina, San Diego, USA), and WES data were filtered as described previously.^39^ The identified *RTN3* variant was verified by Sanger sequencing. RTN3 primer pairs (RTN3 f: 5′‐GCCAGTTGCCGGATTATTCTA‐3′; RTN3 r: 5′‐TGCACAACCTTACGTTCCC‐3′) were designed by IDT browser.

RTN3 amino acid sequences of multiple species were obtained from NCBI (https://www.ncbi.nlm.nih.gov/protein/?term=RTN3). The structure of the human RTN3 protein fragment (Q7RTN4) was downloaded from the AlphaFold database (https://alphafold.ebi.ac.uk/entry/Q7RTN4), and the mutant RTN3 model was constructed using PyMol.

### 5.11 Statistical analysis

Data were subjected to statistical analysis using Graph Pad Prism 8. The mean ± SEM were calculated based on at least three independent experiments. Two‐tailed Student's *t*‐tests and ANOVA were used for two‐group comparisons. Differences were considered statistically significant at *p* ≤ 0.05.

## AUTHOR CONTRIBUTIONS

J.‐Y. J. and L.‐L. F. wrote the draft of the manuscript. J.‐Y. J., H. H., and R. X. designed the project. J.‐Y. J., S.‐H. C., M.‐W. L., and H. H. performed cell and molecular experiments. J.‐Y. J. and Q. W. constructed animal models. Y.‐Q. C., J.‐S. L., and Q. W. enrolled the patient's samples. Y. D. performed animal feeding. H. H. and M.‐W. L. performed the bioinformatic analysis. J.‐Y. J., S.‐H. C., and L.‐L. F. performed genetic analysis. R. X. revised the manuscript. H. H., L.‐L. F., and R. X. supported the project. All authors have read and approved the final manuscript.

## CONFLICT OF INTEREST STATEMENT

The authors declare no conflict of interest.

## ETHICS STATEMENT

This research was approved by the Review Board of Xiangya Hospital of Central South University (animal ethical approval 202103427 and scientific ethical approval 202103427). Written informed consents were obtained from all subjects and their guardians, and they consented to participation in this study and to information publication. This study had been performed in accordance with the ethical standards laid down in the 1964 Declaration of Helsinki and its later amendments.

## Supporting information

Supporting Information

## Data Availability

Experimental data related to the article are available from the corresponding author.

## References

[1] Cantalupo A , Zhang Y , Kothiya M , et al. Nogo‐B regulates endothelial sphingolipid homeostasis to control vascular function and blood pressure. Nat Med. 2015;21(9):1028‐1037. 26301690 10.1038/nm.3934 PMC4692717

[2] Cartier A , Hla T . Sphingosine 1‐phosphate: lipid signaling in pathology and therapy. Science. 2019;366(6463). 10.1126/science.aar5551 PMC7661103 31624181

[3] Yanagida K , Hla T . Vascular and immunobiology of the circulatory sphingosine 1‐phosphate gradient. Annu Rev Physiol. 2017;79:67‐91. 27813829 10.1146/annurev-physiol-021014-071635 PMC5500220

[4] Venkataraman K , Lee YM , Michaud J , et al. Vascular endothelium as a contributor of plasma sphingosine 1‐phosphate. Circ Res. 2008;102(6):669‐676. 18258856 10.1161/CIRCRESAHA.107.165845 PMC2659392

[5] Dantas AP , Igarashi J , Michel T . Sphingosine 1‐phosphate and control of vascular tone. Am J Physiol Heart Circ Physiol. 2003;284(6):H2045‐H2052. 12742827 10.1152/ajpheart.01089.2002

[6] Coussin F , Scott RH , Wise A , Nixon GF . Comparison of sphingosine 1‐phosphate‐induced intracellular signaling pathways in vascular smooth muscles: differential role in vasoconstriction. Circ Res. 2002;91(2):151‐157. 12142348 10.1161/01.res.0000028150.51130.36

[7] Cantalupo A , Di Lorenzo A . S1P signaling and de novo biosynthesis in blood pressure homeostasis. J Pharmacol Exp Ther. 2016;358(2):359‐370. 27317800 10.1124/jpet.116.233205 PMC4959106

[8] Pyne S , Adams DR , Pyne NJ . Sphingosine 1‐phosphate and sphingosine kinases in health and disease: recent advances. Prog Lipid Res. 2016;62:93‐106. 26970273 10.1016/j.plipres.2016.03.001

[9] Wegner MS , Schiffmann S , Parnham MJ , Geisslinger G , Grosch S . The enigma of ceramide synthase regulation in mammalian cells. Prog Lipid Res. 2016;63:93‐119. 27180613 10.1016/j.plipres.2016.03.006

[10] Shi Q , Ge Y , Sharoar MG , et al. Impact of RTN3 deficiency on expression of BACE1 and amyloid deposition. J Neurosci. 2014;34(42):13954‐13962. 25319692 10.1523/JNEUROSCI.1588-14.2014 PMC4198539

[11] Zhou J , Shi Q , Ge YY , et al. Reticulons 1 and 3 are essential for axonal growth and synaptic maintenance associated with intellectual development. Hum Mol Genet. 2023;32(16):2587‐2599. 37228035 10.1093/hmg/ddad085 PMC10407710

[12] Xiang R , Fan LL , Huang H , et al. Increased reticulon 3 (RTN3) leads to obesity and hypertriglyceridemia by interacting with heat shock protein family A (Hsp70) member 5 (HSPA5). Circulation. 2018;138(17):1828‐1838. 29716941 10.1161/CIRCULATIONAHA.117.030718 PMC6392466

[13] Grumati P , Morozzi G , Holper S , et al. Full length RTN3 regulates turnover of tubular endoplasmic reticulum via selective autophagy. eLife. 2017;6. 10.7554/eLife.25555 PMC5517149 28617241

[14] Caldieri G , Barbieri E , Nappo G , et al. Reticulon 3‐dependent ER‐PM contact sites control EGFR nonclathrin endocytosis. Science. 2017;356(6338):617‐624. 28495747 10.1126/science.aah6152 PMC5432029

[15] Fan LL , Du R , Liu JS , et al. Loss of RTN3 phenocopies chronic kidney disease and results in activation of the IGF2‐JAK2 pathway in proximal tubular epithelial cells. Exp Mol Med. 2022;54(5):653‐661. 35596061 10.1038/s12276-022-00763-7 PMC9166791

[16] Song S , Shi Y , Wu W , et al. Reticulon 3‐mediated Chk2/p53 activation suppresses hepatocellular carcinogenesis and is blocked by hepatitis B virus. Gut. 2021;70(11):2159‐2171. 33303565 10.1136/gutjnl-2020-321386

[17] Wang Q , Song X , Du S , et al. Multiple trajectories of body mass index and waist circumference and their associations with hypertension and blood pressure in Chinese adults from 1991 to 2018: a prospective study. Nutrients. 2023;15(3). 10.3390/nu15030751 PMC9919034 36771457

[18] TCS Keller, t, Lechauve C , Keller AS , et al. The role of globins in cardiovascular physiology. Physiol Rev. 2022;102(2):859‐892. 34486392 10.1152/physrev.00037.2020 PMC8799389

[19] Zhu G , Chen H , Zhang W . Phenotype switch of vascular smooth muscle cells after siRNA silencing of filamin. Cell Biochem Biophys. 2011;61(1):47‐52. 21327580 10.1007/s12013-011-9159-7

[20] Kotini MP , van der Stoel MM , Yin J , et al. Vinculin controls endothelial cell junction dynamics during vascular lumen formation. Cell Rep. 2022;39(2):110658. 35417696 10.1016/j.celrep.2022.110658

[21] Feng HZ , Wang H , Takahashi K , Jin JP . Double deletion of calponin 1 and calponin 2 in mice decreases systemic blood pressure with blunted length‐tension response of aortic smooth muscle. J Mol Cell Cardiol. 2019;129:49‐57. 30707993 10.1016/j.yjmcc.2019.01.026 PMC6486848

[22] Tawa H , Rikitake Y , Takahashi M , et al. Role of afadin in vascular endothelial growth factor‐ and sphingosine 1‐phosphate‐induced angiogenesis. Circ Res. 2010;106(11):1731‐1742. 20413783 10.1161/CIRCRESAHA.110.216747

[23] French KJ , Zhuang Y , Maines LW , et al. Pharmacology and antitumor activity of ABC294640, a selective inhibitor of sphingosine kinase‐2. J Pharmacol Exp Ther. 2010;333(1):129‐139. 20061445 10.1124/jpet.109.163444 PMC2846016

[24] Meissner A , Miro F , Jimenez‐Altayo F , Jurado A , Vila E , Planas AM . Sphingosine‐1‐phosphate signalling‐a key player in the pathogenesis of angiotensin II‐induced hypertension. Cardiovasc Res. 2017;113(2):123‐133. 28082452 10.1093/cvr/cvw256

[25] Grumati P , Dikic I , Stolz A . ER‐phagy at a glance. J Cell Sci. 2018;131(17). 10.1242/jcs.217364 30177506

[26] Benjamin EJ , Virani SS , Callaway CW , et al. Heart disease and stroke statistics‐2018 update: a report from the American Heart Association. Circulation. 2018;137(12):e67‐e492. 29386200 10.1161/CIR.0000000000000558

[27] Gerdts E , Sudano I , Brouwers S , et al. Sex differences in arterial hypertension. Eur Heart J. 2022;43(46):4777‐4788. 36136303 10.1093/eurheartj/ehac470 PMC9726450

[28] Theilmeier G , Schmidt C , Herrmann J , et al. High‐density lipoproteins and their constituent, sphingosine‐1‐phosphate, directly protect the heart against ischemia/reperfusion injury in vivo via the S1P3 lysophospholipid receptor. Circulation. 2006;114(13):1403‐1409. 16982942 10.1161/CIRCULATIONAHA.105.607135

[29] Rosen H , Goetzl EJ . Sphingosine 1‐phosphate and its receptors: an autocrine and paracrine network. Nat Rev Immunol. 2005;5(7):560‐570. 15999095 10.1038/nri1650

[30] Chen W , Wu C , Chen Y , et al. Downregulation of ceramide synthase 1 promotes oral cancer through endoplasmic reticulum stress. Int J Oral Sci. 2021;13(1):10. 33753723 10.1038/s41368-021-00118-4 PMC7985500

[31] Yamaji T , Horie A , Tachida Y , et al. Role of intracellular lipid logistics in the preferential usage of very long chain‐ceramides in glucosylceramide. Int J Mol Sci. 2016;17(10). 10.3390/ijms17101761 PMC5085785 27775668

[32] Lee SY , Hong IK , Kim BR , et al. Activation of sphingosine kinase 2 by endoplasmic reticulum stress ameliorates hepatic steatosis and insulin resistance in mice. Hepatology. 2015;62(1):135‐146. 25808625 10.1002/hep.27804

[33] Ishimaru K , Yoshioka K , Kano K , et al. Sphingosine kinase‐2 prevents macrophage cholesterol accumulation and atherosclerosis by stimulating autophagic lipid degradation. Sci Rep. 2019;9(1):18329. 31797978 10.1038/s41598-019-54877-6 PMC6892873

[34] Czopek A , Moorhouse R , Gallacher PJ , et al. Endothelin blockade prevents the long‐term cardiovascular and renal sequelae of acute kidney injury in mice. Sci Transl Med. 2022;14(675):eabf5074. 36516266 10.1126/scitranslmed.abf5074

[35] Zou Y , He W , Wang K , et al. Identification of rare RTN3 variants in Alzheimer's disease in Han Chinese. Hum Genet. 2018;137(2):141‐150. 29356939 10.1007/s00439-018-1868-1

[36] Martinez‐Ramirez M , Madero M , Vargas‐Alarcon G , et al. HDL‐sphingomyelin reduction after weight loss by an energy‐restricted diet is associated with the improvement of lipid profile, blood pressure, and decrease of insulin resistance in overweight/obese patients. Clin Chim Acta. 2016;454:77‐81. 26751808 10.1016/j.cca.2015.12.039

[37] Cantalupo A , Sasset L , Gargiulo A , et al. Endothelial sphingolipid de novo synthesis controls blood pressure by regulating signal transduction and NO via ceramide. Hypertension. 2020;75(5):1279‐1288. 32172624 10.1161/HYPERTENSIONAHA.119.14507 PMC7145736

[38] Zhang N , Zhang Y , Miao W , et al. An unexpected role for BAG3 in regulating PARP1 ubiquitination in oxidative stress‐related endothelial damage. Redox Biol. 2022;50:102238. 35066290 10.1016/j.redox.2022.102238 PMC8783151

[39] Jin JY , Wu PF , Luo FM , et al. GLIS family zinc finger 1 was first linked with preaxial polydactyly i in humans by stepwise genetic analysis. Front Cell Dev Biol. 2021;9:781388. 35087831 10.3389/fcell.2021.781388 PMC8787328

