# Supplementary material for: Reticulon 3 regulates sphingosine‐1‐phosphate synthesis in endothelial cells to control blood pressure
Source: MedComm (2020). 2024 Feb 13;5(2):e480. doi: 10.1002/mco2.480 (PMC10863919; doi:10.1002/mco2.480)
Supplement: Supplementary file 1 — Supporting Information [file MCO2-5-e480-s001.docx]

**Reticulon 3** **regulates sphingosine-1-phosphate synthesis in** **endothelial cells to control** **blood pressure**

Short running title: RTN3 regulates S1P and blood pressure

Jie-Yuan Jin^1, 2, 6^, Si-Hua Chang^2^, Ya-Qin Chen^3^, Meng-Wei Liu^2, 4^, Yi Dong^2^, Ji-Shi Liu^5^, Qin Wang^2,^ ^6^, Hao Huang^1, 2, 6*^, Liang-Liang Fan^2*^, Rong Xiang ^2, 6*^

^1^ Department of Orthopaedics; Microsurgery & Reconstruction Research Center, Xiangya Hospital, Central South University, Changsha, 410000, China;

^2^ School of Life Sciences, Central South University, Changsha, 410000, China;

^3^ Department of Cardiology, the Second Xiangya Hospital of Central South University, Changsha, 410000, China;

^4^ College of Basic Medical, Xinjiang Medical University, Urumqi, 830000, China;

^5^ Department of Nephrology, the Third Xiangya Hospital of Central South University, Changsha, 410000, China;

^6^ National Clinical Research Center for Geriatric Disorders, Xiangya Hospital of Central South University, Changsha, 410000, China.

^*^ Correspondence:

Hao Huang: xyskhuanghao@csu.edu.cn;

Liang-Liang Fan: swfanliangliang@csu.edu.cn;

and Rong Xiang: shirlesmile@csu.edu.cn.

**Supplementary Materials and Methods**

***1. Cell culture and transfection***

Human aortic vascular smooth muscle cells (HA-VSMCs) were obtained from the Cell Bank of Shanghai Institutes for Biological Sciences (Shanghai, China) and cultured in Dulbecco’s modified Eagle’s medium (DMEM). Cells were seed in 6-well plates and transfected with RTN3 siRNA (RiboBio, Guangzhou, China) or plasmids for 24 h to operate CCK8 and flow cytometry, or for 48 h to operate western blot (WB) and co-immunoprecipitation (Co-IP).

***2. Western blot, Co-IP, and confocal***

Proteins were extracted by ristocetin-induced platelet agglutination (RIPA) buffer (Solarbio, Beijing, China), and WB and Co-IP were performed as our previous description^1^. Anti-CANX primary antibody (10427-2-AP, 1:1000), anti-CNN1 primary antibody (24855-1-AP, 1:1000), and anti-CNN2 primary antibody (21073-1-AP, 1:1000) were purchased from Proteintech Company (Wuhan, China). Anti-SPHK1 primary antibody (YN0035, 1:1000), anti-VCL primary antibody (YT4882, 1:1000), and anti-α-SMA primary antibody (YT5053, 1:1000) were purchased from ImmunoWay Biotechnology Company (Suzhou, China). Anti-CERS1 primary antibody (sc-293497, 1:100) was purchased from Santa Cruz Biotechnology Incorporated (Santa Cruz, USA). Ceramide antibody (C8104, 1:100) purchased from Merck KGaA (Darmstadt, Germany) was used for confocal^2^.

***3. ELISA and NO assay***

S1P was tested in the mouse plasma isolated from blood sampled by eyeball extirpating, in the aortic tissue homogenate of mice dissolved in PBS, and in the supernatant from cells cultured in complete medium for 48 h, using S1P ELISA assay kits (Mlbio, Shanghai, China). Standard curve was drew based on the measurement of S1P standards with 10 nmol/L, 50 nmol/L, 100 nmol/L, 200 nmol/L, 400 nmol/L, and 800 nmol/L. 50 μL sample was mixed with 100 μL horse radish peroxidase and incubated at 37 ℃ for 60 min. 50 μL substrate A and 50 μL substrate B were added into the mixture and incubated at 37 ℃ in the dark for 15 min. After 50 μL Stop Buffer being rapidly appended, samples were measured in the 450 nm absorbance.

NO assay kit (Beyotime, Shanghai, China) was used to detect the NO content in the culture supernatant. 1 M Na_2_NO_2_ standard solution was diluted to 2 μM, 5 μM, 10 μM, 20 μM, 40 μM, 60 μM, and 80 μM solutions. 5 μL 2 mM NAPDH, 10 μL FAD, and 5 μL Nitrate Reductase were added in 60 μL samples, and incubated in 37 ℃ for 30 min. And then 10 μL LDH buffer and 10 μL LDH were also added into the mixture to incubate in 37 ℃ for 30 min. Finally, 50 μL Griess Reagent I buffer and 50 μL Griess Reagent II buffer were added, with incubation at room temperature for 10 min, and their absorbances at 540 nm wavelength were measured.

Cells number was counted by cell counter. Cells were collected, dissolved in PBS, and lysed by freeze-thaw cycles. The lysates were used to detected ceramide and cGMP, severally using ceramide and cGMP ELISA assay kits (Mlbio, Shanghai, China). The concentrations of ceramide standards included 0 μmol/L, 10 μmol/L, 20 μmol/L, 40 μmol/L, 80 μmol/L, and 160 μmol/L. While cGMP standards concentrations were respectively 0.375 nmol/L, 0.75 nmol/L, 1.5 nmol/L, 3 nmol/L, 6 nmol/L, and 12 nmol/L.

***4. CCK8 and flow cytometry***

Cells were seed in 96-well plates, and CCK8 was performed using CCK8 assay kit (Solarbio, Beijing, China). Flow cytometry was executed by Cell Center, Central South University (Changsha, China). Annexin V-FITC (Beyotime, Shanghai, China) was added into cell samples, and then propidium iodide (PI; Beyotime, Shanghai, China) was added to test cell apoptosis. 7-AAD (BD Biosciences, New Jersey, China) was used to detect cell cycle.

***5. unique identifier (UID)-mRNA sequencing***

UID RNA-seq experiment and high through-put sequencing and data analysis were conducted by Seqhealth Technology (Wuhan, China). 2 μg total RNAs were used for stranded RNA sequencing library preparation using KC-DigitalTM Stranded mRNA Library Prep Kit for Illumina (Catalog NO. DR08502; Seqhealth, Wuhan, China) following the manufacturer’s instruction. The kit eliminates duplication bias in PCR and sequencing steps, by using unique molecular identifier (UMI) of 8 random bases to label the pre-amplified cDNA molecules. The library products corresponding to 200-500 bps were enriched, quantified and finally sequenced on Novaseq 6000 sequencer (Illumina, San Diego, USA) with PE150 model.

**References**

1. Xiang R, Fan LL, Huang H, et al. Increased Reticulon 3 (RTN3) Leads to Obesity and Hypertriglyceridemia by Interacting With Heat Shock Protein Family A (Hsp70) Member 5 (HSPA5). *Circulation*. 2018;138(17):1828-1838.

2. Li T, Ying L, Wang H, et al. Microcystin-LR induces ceramide to regulate PP2A and destabilize cytoskeleton in HEK293 cells. *Toxicol Sci*. 2012;128(1):147-57.


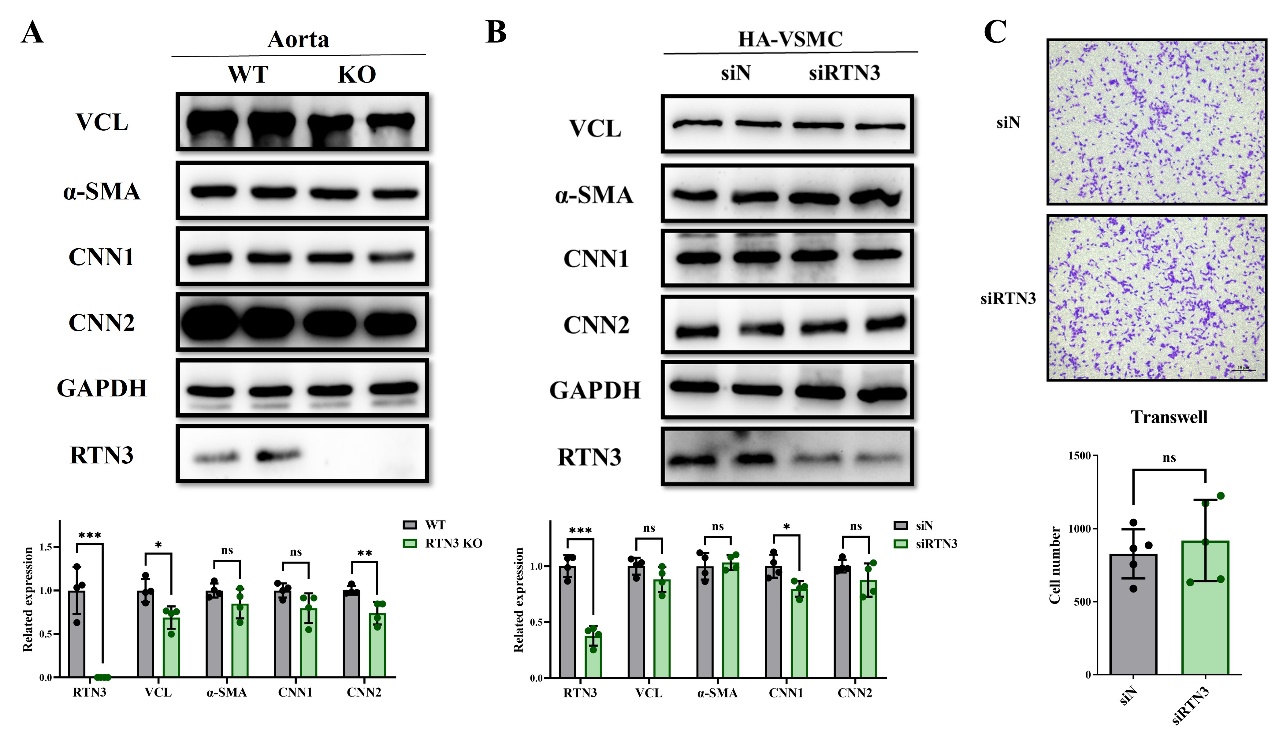


**Supplemental figure 1. Reduced RTN3 does not seemingly affect the skeleton proteins and migration of vascular smooth muscle cells.** (A and B) The expression of VCL, α-SMA, CNN1, CNN2, GAPDH, and RTN3 in the vascular smooth muscle of wild-type (WT) mice and RTN3-null mice (A), and HA-VSMCs with/without siRTN3 transfection (B). (C) Cell migration of HA-VSMCs transfected with siRTN3 and controls was detected by transwell.

**
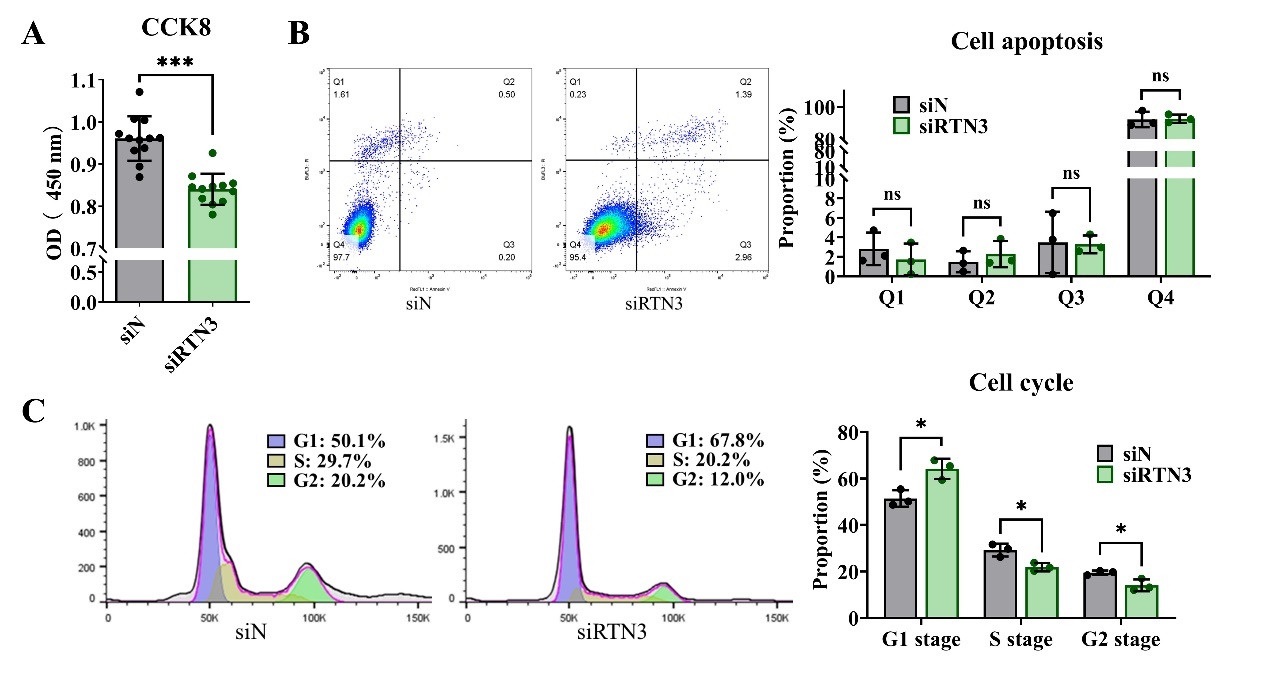
**

**Supplemental figure 2. Reduced RTN3 restrains cell viability in HUVECs.** (A) Cell activity in HUVECs transfected with siRTN3 and controls detected by CCK8. (B and C) Cell apoptosis (B) and cell cycle in HUVECs with/without siRTN3 transfection were detected by flow cytometry.


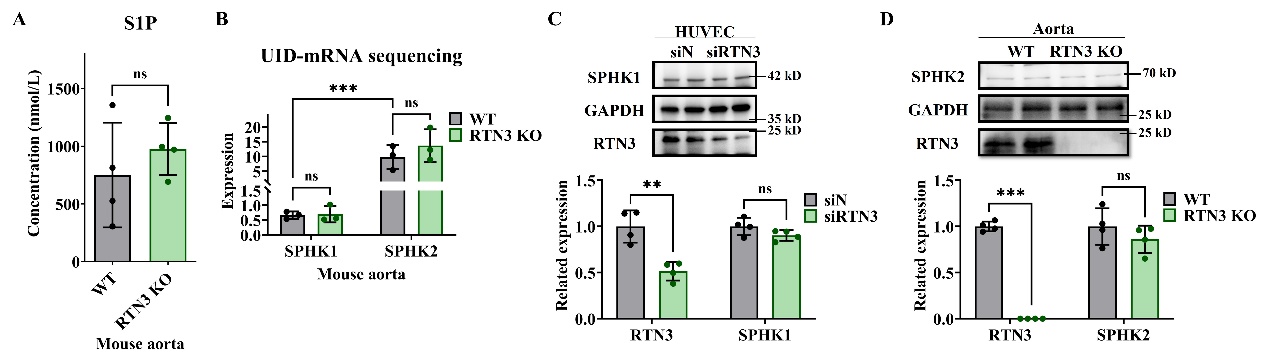


**Supplemental figure 3. The expression of S1P, SPHK1, and SPHK2 in mouse aortas and HUVECs.** (A) Sphingosine-1-phosphate (S1P) levels of aortas in wild-type (WT) mice and RTN3 KO mice. (B) The expressions of SPHK1 and SPHK2 in mouse aortas were detected by UID-mRNA sequencing. (C) The expression of RTN3 and SPHK1 in HUVECs with/without RTN3 siRNA transfection. (D) The expression of RTN3 and SPHK2 of aortas in WT mice and RTN3 KO mice.


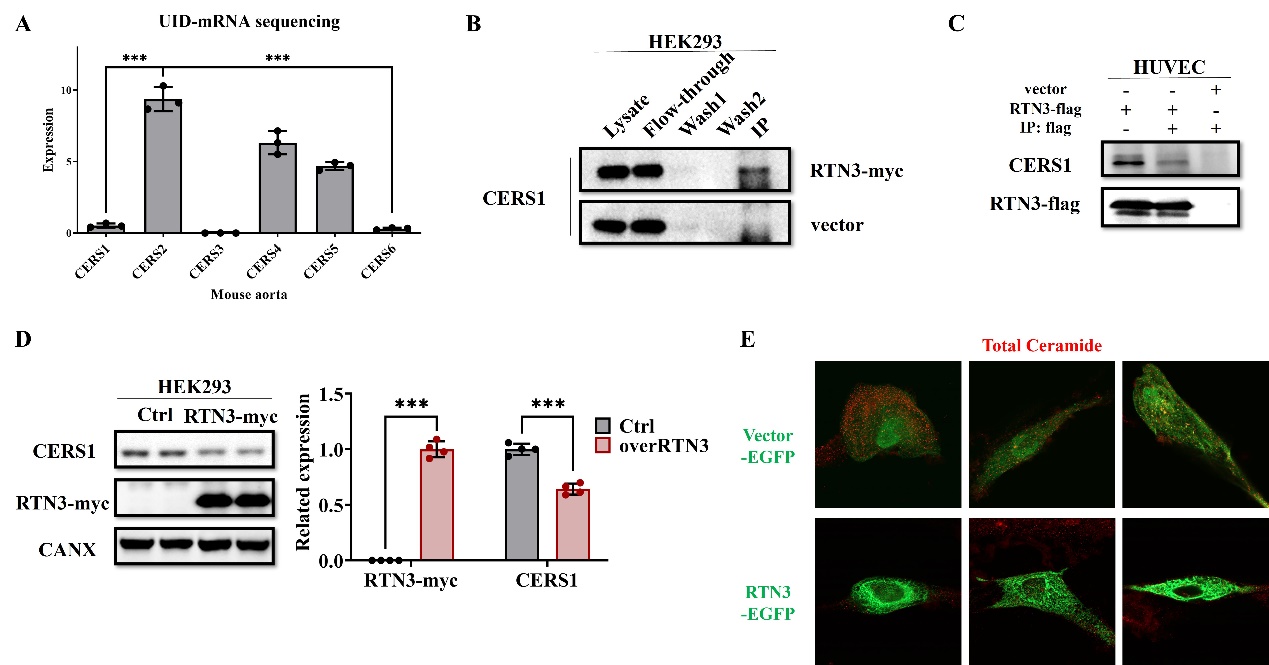


**Supplemental figure 4. RTN3 interacts with CERS1 to play a negative role in ceramide synthesis.** (A) The expressions of CERS1-6 in mouse aortas were detected by UID-mRNA sequencing. (B and C) Coimmunoprecipitation (Co-IP) confirmed that RTN3 can interact with CERS1 in HEK293 cells (B) and HUVECs (C). (D) The protein levels of RTN3-myc and CERS1 in the HEK293 cells with RTN3 plasmid transfection and controls. (E) The levels of total ceramide (red) and RTN3-EGFP (green) were detected by confocal microscopy.
